# Complete Genome Sequence of *Staphylococcus xylosus* HKUOPL8, a Potential Opportunistic Pathogen of Mammals

**DOI:** 10.1128/genomeA.00653-14

**Published:** 2014-07-24

**Authors:** Angel Po Yee Ma, Jingwei Jiang, Hein Min Tun, Nathalie France Mauroo, Chan San Yuen, Frederick Chi-Ching Leung

**Affiliations:** a School of Biological Sciences, the University of Hong Kong, Hong Kong SAR, China; b Bioinformatics Center, Nanjing Agricultural University, Nanjing, China; c Department of Pathology, the University of Hong Kong, Hong Kong SAR, China; d Clinical Laboratory, Veterinary Center, Ocean Park Corporation, Hong Kong SAR, China

## Abstract

We report here the first complete genome sequence of *Staphylococcus xylosus* strain HKUOPL8, isolated from giant panda feces. The whole genome sequence of this strain will provide an important framework for investigating the genes responsible for potential opportunistic infections with this species, as well as its survival in various environments.

## GENOME ANNOUNCEMENT

*Staphylococcus xylosus* is a ubiquitous Gram-positive bacterium that was initially isolated from human skin (1) and forms part of the commensal skin flora on humans and mammals (2). It has traditionally been regarded as an apathogenic member of the coagulase-negative staphylococci and is versatile for many applications including fermentation of meat and dairy products (3, 4). However, some virulent strains may participate in opportunistic infections in humans and mammals (5, 6). We report here the complete genome of *S. xylosus* strain HKUOPL8, isolated in 2012 from fresh fecal matter from a healthy giant panda (*Ailuropoda melanoleuca*).

Whole-genome sequencing of strain HKUOPL8 was performed with 454 pyrosequencing technology (7). *De novo* shotgun and 8-kb paired-end libraries were constructed and sequenced with the 454 GS Junior platform (454 Life Sciences, Branford, CT). A total of 69,853,635 bp in 151,536 reads from the shotgun library and 128,836,151 bp in 358,654 reads from the 8-kb paired-end library were assembled with the 454 Newbler software (454 Life Sciences, Branford, CT), yielding a total of 140 contigs with an *N*_50_ value of 76,932 bases. Gaps between contigs were filled by Sanger sequencing of PCR products using an ABI 3130XL capillary sequencer; subsequent assembly was performed with SeqMan software (DNASTAR). The open reading frames and rRNA and tRNA genes were annotated by the NCBI Prokaryotic Genome Automatic Annotation Pipeline (PGAAP) (8). Functional classification was performed by aligning predicted proteins to the clusters of orthologous groups (COGs) database (9). All predicted genes were compared to a non-redundant protein database in NCBI using BLASTx (10), with *E* values of 1×10^−5^ and filtering of 20% match identity and 90% alignment length. Metabolic pathways were analyzed by a bi-directional best-hit method on the KEGG web server (11).

The complete genome of *S. xylosus* strain HKUOPL8 contains one circular chromosome of 2,836,901 bp with a G+C content of 32.8%, which is similar to other staphylococci (12), and one circular plasmid of 30,062 bp in size. Chromosome sequence annotation revealed 2,538 coding sequences (CDSs), of which 50% were connected to the COGs. A total of 66 RNA genes, including 13 rRNA and 53 tRNA genes, were found on the chromosome. Approximately 88.8% of the CDSs were assigned a KEGG orthologous number and were involved in 161 predicted metabolic pathways. Gene clusters such as *ureABCEFGD* (the urease gene operon) and SCC*cap1* (staphylococcal cassette chromosome type 1 capsule genes) were identified, along with virulence genes including EF0577 and *mtsABC*, both associated with iron and manganese transport (ABC transporters). These elements may increase virulence and enable strain HKUOPL8 to cause opportunistic infections in mammals when the host immune system is below par. Also, mucoid production from type 1 capsular genes may render strain HKUOPL8 more robust to survive in harsh environments by resisting phagocytosis (13).

### Nucleotide sequence accession numbers.

The complete genome sequence of *S. xylosus* strain HKUOPL8 has been deposited in GenBank under the accession no. CP007208 for the chromosome and CP007209 for the plasmid. The version described in this paper is the first version.
